# Spatial and temporal patterns of agrometeorological indicators in maize producing provinces of South Africa

**DOI:** 10.1038/s41598-022-15847-7

**Published:** 2022-07-15

**Authors:** Christian Simanjuntak, Thomas Gaiser, Hella Ellen Ahrends, Amit Kumar Srivastava

**Affiliations:** 1 Institute of Crop Science and Resource Conservation, University of Bonn, Katzenburgweg 5, 53115 Bonn, Germany; 2 Department of Agricultural Sciences, University of Helsinki, Koetilantie 5, 00014 Helsinki, Finland

**Keywords:** Climate change, Climate and Earth system modelling, Climate-change impacts, Climate-change mitigation, Climate sciences

## Abstract

Climate change impacts on maize production in South Africa, i.e., interannual yield variabilities, are still not well understood. This study is based on a recently released reanalysis of climate observations (AgERA5), i.e., temperature, precipitation, solar radiation, and wind speed data. The study assesses climate change effects by quantifying the trend of agrometeorological indicators, their correlation with maize yield, and analyzing their spatiotemporal patterns using Empirical Orthogonal Function. Thereby, the main agrometeorological factors that affected yield variability for the last 31 years (1990/91–2020/21 growing season) in major maize production provinces, namely Free State, KwaZulu-Natal, Mpumalanga, and North West are identified. Results show that there was a significant positive trend in temperature that averages 0.03–0.04 °C per year and 0.02–0.04 °C per growing season. There was a decreasing trend in precipitation in Free State with 0.01 mm per year. Solar radiation did not show a significant trend. Wind speed in Free State increased at a rate of 0.01 ms^−1^ per growing season. Yield variabilities in Free State, Mpumalanga, and North West show a significant positive correlation (r > 0.43) with agrometeorological variables. Yield in KwaZulu-Natal is not influenced by climate factors. The leading mode (50–80% of total variance) of each agrometeorological variable indicates spatially homogenous pattern across the regions. The dipole patterns of the second and the third mode suggest the variabilities of agrometeorological indicators are linked to South Indian high pressure and the warm Agulhas current. The corresponding principal components were mainly associated with strong climate anomalies which are identified as El Niño and La Niña events.

## Introduction

Climate change has become enormous challenge and it has impacted nearly every aspect of human life, for example, health^1^, socio-economy^2^, ecosystems^3^, and global food security^4^. The consequences are expected to escalate in conjunction with constant climate change. According to the IPCC 2021^5^ report, global warming is very likely to reach 1.8 °C under the very low greenhouse gas emission scenario between 2081 and 2100. This can lead to changing precipitation patterns and intensities, rising sea levels, and more frequent occurrences of extreme climate events in many regions across the globe. The number of studies providing evidence on the regional impacts of climate change in Africa is increasing. Huang, et al.^6^ reported a shift in regional climate patterns for Southern Africa, where semi-arid regions are being transformed into arid regions. Moreover, the significant decline of total precipitation with 31.13 mm per decade in Central Africa could lead to more frequent droughts in the future^7^.

South Africa is recognized as being the country with the highest per capita emission among developing countries because the domestic economy heavily depends on coal resources to produce energy^8^. A previous study on temperature trends in South Africa revealed that the annual mean temperature has significantly increased by 0.13 °C per decade between 1960 and 2003^9^. It is projected that under a 1.5 °C Global Warming Level (GWL), South Africa will be experiencing a reduction in precipitation of approximately 0.4 mm per day. Temperature will increase, in particular during the September–October–November season^10^. If this trend continues, it will lead to drastic reductions in the potential agricultural production around 15% up to 50% by 2080^11^. Farmers in South Africa have already acknowledged the significant climate change and are shifting their production to more drought tolerant varieties^12^.

Maize is an important cereal crop worldwide because it is considered a staple food in many countries including South Africa. The maize production in South Africa is realized by both commercial and small-scale farmers. They are responsible for 98% and 2% of the total production, respectively. The majority of maize production is centralized in Free State, KwaZulu-Natal, Mpumalanga, and North West regions where in 2019 maize yields accounted for 38%, 6%, 25%, and 15% of the total national production, respectively^13,14^. In addition, a recent study reported that the total maize production in South Africa rose from 1.68 million tons in 1935 to 12.2 million tons in 2015, despite a decline in the production area of 35.7%^15^.

In order to assist mitigation and adaptation planning and predict future impacts of climate change on maize production in South Africa, it is necessary to not only understand the historical patterns and trends of agrometeorological indicators but also their statistical relation with maize yield. Moreover, with the topography characteristic in South Africa which are plateau, coastal line, and mountains, it is important to describe the climate variability on different elevation^16^. Past research studies utilized climate reanalysis data which were not tailored for assessing impacts on agricultural production. Thus, study results could be misinterpreted especially with respect to the agricultural production sector. For this study, we employed the reanalysis climate data set AgERA5 which has been developed for being used in agricultural and agroecological studies^17^. The data set covers the period from January 1979 and it is frequently updated close to real-time. It is based on hourly ECMWF ERA5 data at surface level and has a spatial resolution of 0.1°^18^. Because AgERA5 has only recently been released, not many researchers have associated the AgERA5 dataset into their work, especially in investigating maize production in South Africa. Furthermore, there is no report available that explicitly evaluates changes and impacts of climate variabilities (spatiotemporal) during the maize growing season data across different provinces. Therefore, the objectives of this study were to (1) identify and quantify the trends in agrometeorological indicators for the period 1990–2021, (2) investigate the statistical relation between agrometeorological indicators and maize yields, and (3) identify changes in spatiotemporal patterns during the maize growing season.

## Materials and methods

### Study area description

**Figure 1:**
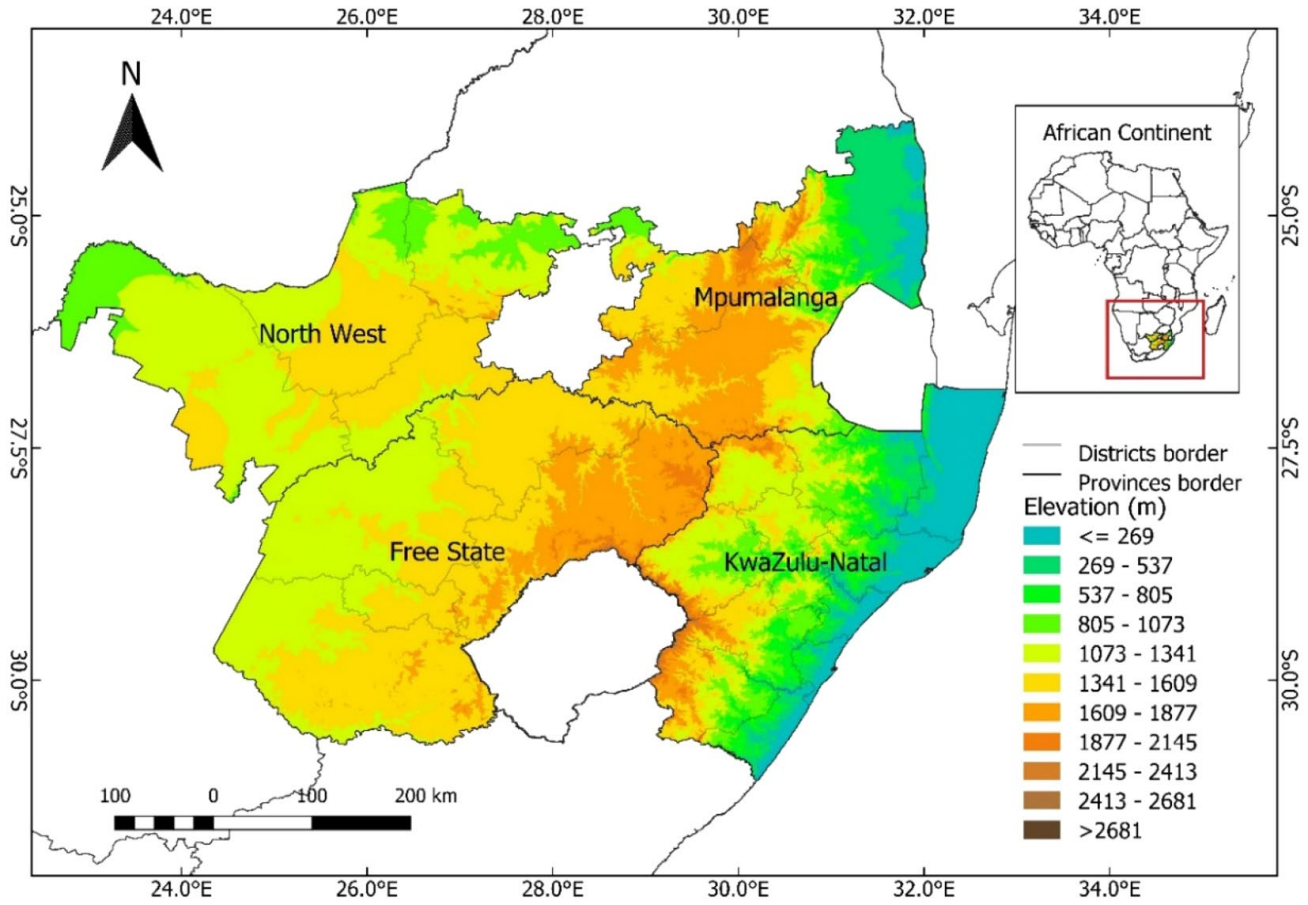
Map of the study area. South African provinces (Free State, KwaZulu-Natal, Mpumalanga, and North West) are shown. (The figure was generated by QGIS 3.16.7-Hannover software, https://www.qgis.org/en/).

South Africa is located at the southern tip of Africa and lies between latitudes -35°S and -22°N, and longitudes 16° W and 33° E spreading over an area of 1,221,037 km^2^. It is surrounded by the Indian Ocean in the east and the Atlantic Ocean in the west. Only conditions in the major maize production provinces of South Africa, namely Free State, KwaZulu-Natal, Mpumalanga, and North West provinces (Fig. 1) are considered. **Free State** has an administrative zone area of 130,081 km^2^ and it is located in east-central South Africa. The climate is typically hot semi-arid. **KwaZulu-Natal** occupies an administrative area of 93,421 km^2^ and it is located in southeastern South Africa with the climate for most areas being classified as humid subtropical. **Mpumalanga,** with a surface area of 76,831 km^2^, located in north-eastern South Africa, mostly has a humid subtropical climate. **North West** is located in north-central South Africa. It has a surface area of 105,028 km^2^. The hot semi-arid climate is prevalent in this region.

### Research data

The agrometeorological data were retrieved from the reanalysis AgERA5^18^ climate data set which is available at daily time step and at 0.1^o^ × 0.1° spatial resolution spanning from 1990 to 2021 over the study area (Table 1). Data are statistically aggregated to 24 h values using aggregation schema that have been developed for eight different longitudinal zones^19^.

**Table 1:** Agrometeorological variables of the AgERA5 data set used in this study.

Variable	Unit	Abbreviation
Temperature at 2 m above the surface	^o^C	T
Precipitation	mm day^−1^	PP
Solar radiation flux	MJ m^−2^ day^−1^	SR
Wind speed at 10 m above the surface	M s^−1^	WS

For our study, we distinguished between ‘annual’ and ‘growing season’ of maize data sets. The growing season of maize begins in October and the maturity date can be expected in March of the following year, depending on the sowing time^20–24^. Historical maize yield data for each province were obtained from the Statistics and Economic Analysis of Agriculture, Forestry and Fisheries Department South Africa (Figures S1, S2). A digital elevation model (DEM) was obtained from Open Topography as part of the SRTM15Plus dataset. This dataset has a sampling interval of 15 arc sec (approximately 500 × 500 m pixel size at the equator)^25^. To identify the distribution of maize fields in the study regions, the 100 m land cover (LC) map for the year of 2015 and 2019 from Copernicus Global Land Service (CGLS) were used. In this study, cropland is assumed mainly being used to cultivate maize. The reason behind this is, as described in the Validation CGLS report, the cropland of CGLS map is identified as a temporary crop followed by harvest and a bare soil period^26^. In the interest regions, the typical rainfed maize system has 3–4 months fallow period after harvesting^22^. In addition, the study regions are the main regions for maize production in South Africa^13^.

### Data analysis

To detect monotonic agrometeorological trends over time, the Mann Kendall test was employed. The Mann Kendall test is well known as a non-parametric statistical which is suitable for non-normally distributed data and it is less affected by extreme outliers in the series^27^. This test is widely applied by environmental studies because it is robust and can cope with missing values. We tested for serial autocorrelation using the autocorrelation function (ACF) and the partial autocorrelation function (PACF). The results showed no significant serial correlation. Thus, we carried out the Mann Kendall test^28,29^. The original Mann Kendall test statistic (S) is given using the following equation:

1 $$S=\sum_{k=1}^{n-1}\sum_{j=k+1}^{n}sgn\left({x}_{j}-{x}_{k}\right)$$

where,

2 $$sgn\left({x}_{j}-{x}_{k}\right)=\left\{\begin{array}{c}1 if {x}_{j}>{x}_{k}\\ 0 if {x}_{j}={x}_{k}\\ -1 if {x}_{j}<{x}_{k}\end{array}\right.$$

the time series x_1_, x_2_, x_3_…x_n_ represents n data points. ${x}_{j}$ and ${x}_{k}$ are the climate data value. It follows the logical order in the month of $j$ and $k$ ($j>k$). Because of n > 10, we computed the variance of S as follow:

3 $$VAR\left(S\right)=\frac{1}{18}\left[n\left(n-1\right)\left(2n+5\right)-\sum_{p=1}^{q}{t}_{p}({t}_{p}-1)(2{t}_{p}+5)\right]$$

where q is the number of tied groups (the sample data have the same value) and t_p_ is the number of data points in the p^th^ tied group. Afterward, Z is used to indicate the trend’s significance level, using the following equation:

4 $$Z_{{mk}} \left\{ {\begin{array}{*{20}l} { = \frac{{S - 1}}{{\left[ {VAR\left( S \right)} \right]^{{1/2}} }}} \hfill & {if\,S > 0} \hfill \\ { = 0} \hfill & {if\,S = 0} \hfill \\ { = \frac{{S + 1}}{{\left[ {VAR\left( S \right)} \right]^{{1/2}} }}} \hfill & {if\,S < 0} \hfill \\ \end{array} } \right.$$

Given a confidence level α, the upward trends are indicated by the positive value of Z, if the absolute value of Z is greater than Z_1-α_. The downward trend is shown by a negative value of Z if the absolute value of Z is greater than Z_1-α/2_.

The magnitude of the trend was determined using the non-parametric Sen’s slope approach^30^. The magnitude can be estimated using the following equation^31^:

5 $$\beta =Median\left(\frac{{x}_{j}-{x}_{i}}{j-i}\right),\mathrm{ for }(1 \le i < j \le n)$$

where β is Sen’s slope estimator, x donates the variables, n is the number of data, and i, j are indices. β > 0 represents an increasing trend, in contrast, β < 0 denotes a decreasing trend.

Linear regression was applied to those variables that showed a significant trend in all regions. The slope of the regression line was considered as an indicator for positive or negative long-term trends^29^.

To investigate the relationship of agrometeorological variation with respect to the elevation, we performed a linear regression analysis of extracted data points occupying in the study area. The agrometeorological data for this analysis was based on the maize growing season (see section “Research data”). The rectangle grid 0.1*0.1 degree was overlayed in each raster layer for the agrometeorological data and the elevation map. Centroid of each rectangle grid represents as a data point. The total extracted data points from Free State, KwaZulu-Natal, Mpumalanga, and North West were 1217, 953, 786, and 1048, respectively. Similar procedure was performed using the rectangle grid 0.01*0.01 degree for the land cover and the elevation map, to investigate the location of maize field at different elevation level. The total data point (see section “Research data”) was extracted from Free State, KwaZulu-Natal, Mpumalanga, and North West were 120,839, 86,932, 69,885, and 95,943, respectively.

To investigate the statistical relation between the temporal variability of agrometeorological data during the maize growing season with maize yields, we performed a Pearson correlation. Since maize yields in different regions are highly influenced by agro-management, technology improvement, and socio-economic factors, we de-trended the maize yield^32^. We also de-trended agrometeorological data that showed a significant trend using pracma package in R^33^. The assumptions for Pearson correlation were tested prior to analysis. No serial correlation was detected and the normality assumption was satisfied. If required, box-cox transformation was performed. Thus, our results should be interpreted with caution. The correlation coefficient can be calculated using the following equation:

6 $$r=\frac{{\sum }_{i=1}^{n}({x}_{i}-\overline{x })({y}_{i}-\overline{y })}{\sqrt{{\sum }_{i=1}^{n}{({x}_{i}-\overline{x })}^{2}{({y}_{i}-\overline{y })}^{2}}}$$

where r is the correlation coefficient of agrometeorological data and yield data, *x*_*i*_ is the daily mean of agrometeorological variables in the ith season, *y*_*i*_ is the yield value in the ith season, and n is the sample size.

To investigate the dominant spatiotemporal patterns of the agrometeorological data, we applied an EOF (Empirical Orthogonal Functions) analysis, also referred to as PCA (Principal Component Analysis). The method analyzes the changes in oscillations over time and extracts the dominant pattern (“leading modes”) that contribute the strongest to the total variability^34^. In this particular analysis, only daily grid data during maize growing period data was used (see section “Research data”). Because the growing season of maize in South Africa extends from the end of a particular year to the beginning of the following year. The growing season period was identified as the year when the maize was sown, for instance: growing season 1990/1991 is referred to as 1990.We carry out this naming to all growing seasons.

We performed stepwise approach to derive EOF and its corresponding principal coefficient (PC) using the Climate Data Operators (CDO) version 1.9.4rc1^35^. The EOF partitions the variance into space and modes (EOFs) and time (principal components). The opted procedure is as follows:

- In the first step, each agrometeorological variable (T, PP, SR, and WS) was extracted based on the growing season of maize and the monthly anomaly data was calculated (*cdo selseason* and *cdo ymonsub operator*);
- In the second step, The EOFs (*cdo eof operator*) were calculated with the environments of the algorithm for eigenvalue calculation, the weight mode, and Frobenius norm set to default mode (*export CDO_SVD_MODE* = *jacobi*, *export CDO_WEIGHT_MODE* = off, and *FNORM_PRECISION* = *1e-12*, respectively). The maximum integer number was modified to 100 (*export MAX_JACOBI_ITER* = *100*). The results are the eigenvectors *ej, j* = *1,…p.* These eigenvectors are spatial patterns or “structures” of the major factors that explain the amount of variance of the time series of the sample;
- Further, to obtain the percentage variance explained by each pattern, the quotient of the eigenvalue and the total variance of the input data were calculated;
- In the last step, the corresponding principal coefficients (PC) of EOFs were obtained (*cdo eofcoeff operator*).

Maps shown in this manuscript were generated using the open-source software QGIS 3.16.7-Hannover software (https://www.qgis.org/en/).

## Results

### Agrometeorological trend

Based on the daily temperatures (T) AgERA5 over the period 1990 to 2020, T significantly increased in all regions, at an average rate of 0.03–0.04 °C per year. Similarly, if limiting the data set to the maize growing season, T significantly increased over time with the magnitude being lowest for KwaZulu-Natal and Mpumalanga at 0.02 °C per growing season (Table 2). The predominant T upward trend for the annual and growing season data set is shown in Figures S3 and S4 of the supplementary material, respectively. Patterns and trends of T in maize growing season T (Figure S4) are similar to those of annual T (Figure S3). A large positive temperature deviation can be observed in 2015/2016 (Figure S4). Annual precipitation (PP) showed a significant downward trend, particularly in the Free State province. Although there was no significant reduction in PP for the other regions, negative slopes suggested decreasing PP. No significant trend was detected for solar radiation (SR) data. This is in contrast with annual wind speed (WS), for which increasing trends were found at Free State, KwaZulu-Natal, and North West provinces with a magnitude of 0.004, 0.002, 0.003 m s^−1^ year^−1^ respectively. For the growing season data set, only Free State showed an upward trend in WS with 0.01 m s^−1^ per growing season.

**Table 2:** Summary statistic of Mann Kendall test (Z) and Sen’s Slope estimator (β) for annual (1990–2020) and maize growing season (1990/91–2020/21).

Time	Agrometeorological	Free State		KwaZulu-Natal		Mpumalanga		North West	
		Z	β	Z	β	Z	β	Z	β
Annual	T	3.64**	0.04	3.26**	0.03	3.26**	0.03	2.82**	0.04
	PP	− 2.14*	− 0.01	− 1.29	− 0.01	− 0.61	− 0.01	− 1.66	− 0.01
	SR	1.70	0.01	0.82	0.01	0. 23	0	0.82	0.01
	WS	2.75**	0.004	1.97*	0.002	0.61	0	2.04*	0.003
Growing season	T	3.06**	0.04	2.82**	0.02	2.04**	0.02	2.69**	0.04
	PP	− 1.73	− 0.02	− 1.05	− 0.01	− 0.30	0	− 0.91	− 0.01
	SR	1.05	0.02	0.71	0.01	− 0.03	0	0.31	0
	WS	2.31*	0.01	1.29	0	0.14	0	1.83	0

**Significant level at α < 0.01; *significant level at 0.01 < α ≤ 0.05.

The beta coefficient (β) indicates changes per year and changes per growing season. See Table 1 for abbreviations of agrometeorological data.

### Topography and cropland

A negative relationship between temperature and elevation was found for all four regions (Figure S5a), where the decreasing magnitude of temperature was associated with high altitude (r^2^ > 0.88). In contrast with precipitation, Fig. S5b shows that there was a positive relationship with elevation. High precipitation reaching 10 mm day^−1^ was spread out above 500 m above sea level, especially KwaZulu-Natal and Mpumalanga. For solar radiation (Fig. S5c), it appears that the slight relationship of low solar radiation magnitude (17–25 MJ m^−2^ day^−1^) extended in all elevation level of KwaZulu-Natal and Mpumalanga. On the other hand, the solar radiation in Free State and North West has a negative relationship, where its magnitude was dominant at elevation of 1000–2000 m ranging from 22 to 27 MJ m^−2^ day^−1^. For wind speed (Fig. S5d), there was no significant relationship with elevation in Free State, Mpumalanga, and North West. When comparing with wind speed in KwaZulu-Natal where is located on the east coast of South Africa, it shows that the majority of high intensity wind speed was located at 0–500 m above sea level.

**Figure 2:**
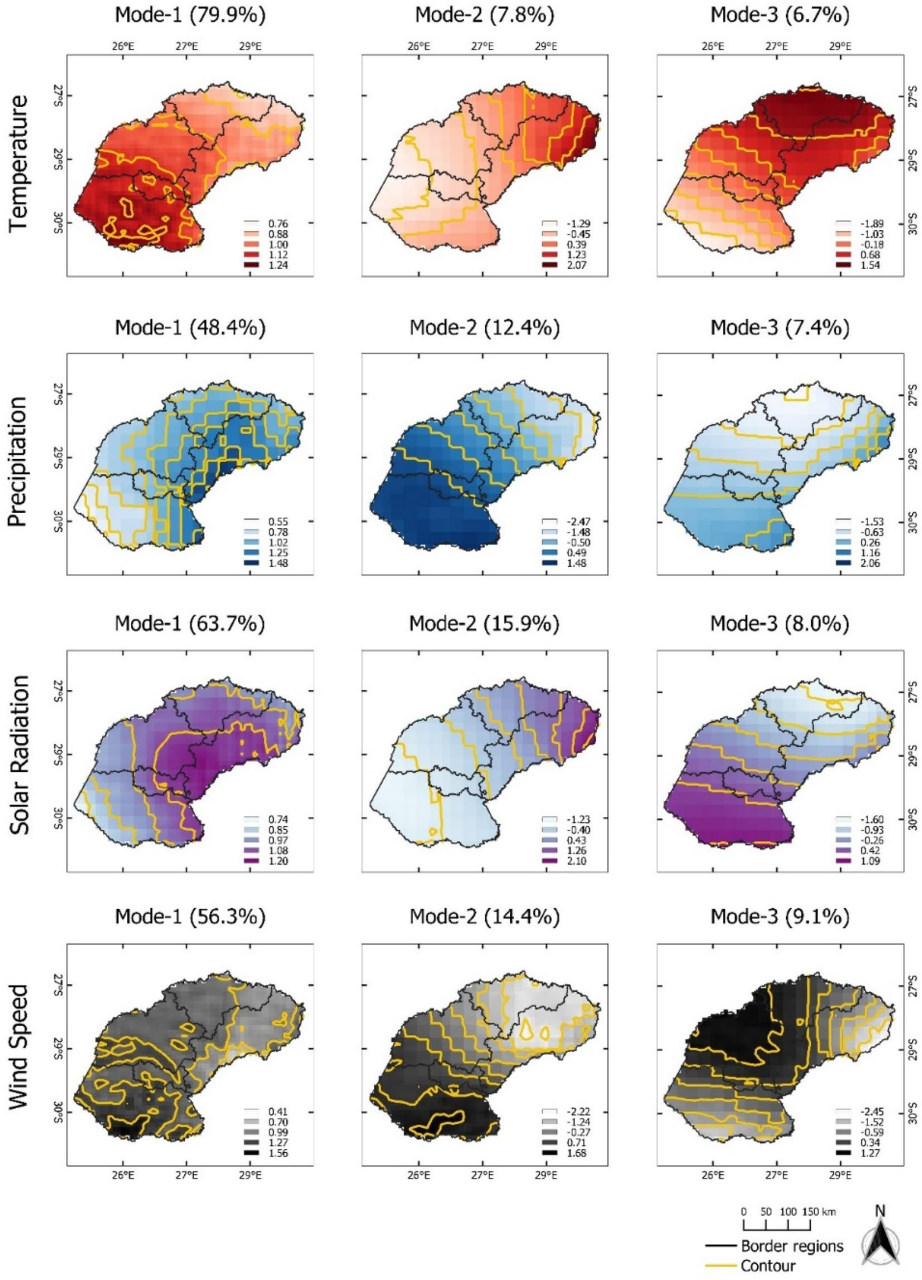
Map pattern of factor loadings for the first three modes (columns) derived from an EOF analysis for temperature, precipitation, solar radiation, and wind speed anomalies (rows) observed in the **Free State** province during the maize growing season of 1990/90–2020/21. Data for the maize growing season was used (cf. “Research data” section). Contour (yellow lines) are used to highlight spatial patterns in agrometeorological data.

Figure S6 shows the distribution of cropland (maize) spread throughout the regions. It is located at all ranges of elevation level especially in Mpumalanga and North West (Figure S7). For Free State and Kwazulu-Natal, the cropland is spotted at elevation 2500 m above sea level. These findings provide the evidence that maize field (crop land) could be found all over the interest regions and this enables us to examine the impact of agrometeorological variables on maize yield at the regional scale.

### Statistical relation between agrometeorological data and maize yields

Pearson correlation coefficients indicate a significant negative correlation between yields with T, SR, and WS, but a positive correlation with PP (Table 3). For the Free State province, all agrometeorological variables showed significant correlations with maize yield. The highest correlation was observed for PP and SR (0.60), and the lowest for WS (0.43). Agrometeorological data observed in the Kwazulu-Natal province were not significantly correlated with maize yields. For the Mpumalanga province, a positive correlation of PP was acknowledged at 0.43, at the same time the negative correlation of SR was 0.48. In the last regions, North West, a strong correlation was identified on PP and SR, not to mention both T and WS had a moderate negative correlation at 0.65 and 0.43, respectively.

**Table 3:** Pearson correlation coefficient (r) between growing season data of agrometeorological variables and maize yield (see Table 1 for abbreviations).

Agrometeorological	Free state	Kwazulu-Natal	Mpumalanga	North West^+^
T	− 0.51**	− 0.13	− 0.28	− 0.65**
PP	0.60**	− 0.02	0.43*	0.74**
SR	− 0.60**	− 0.22	− 0.48**	− 0.73**
WS	− 0.43*	0.24	− 0.12	− 0.43*

**Significant level at α < 0.01; *significant level at 0.01 < α ≤ 0.05; ^+^yield data was transformed using boxcox.

### Spatial and temporal patterns

To distinguish spatiotemporal patterns in agrometeorological time-series data (spatial maps showing coherent variations of time series data), we displayed the first three dominant modes of EOF which represent a spatial pattern (spatial modes of variability) and its corresponding principal components (PC) as a time signature (how patterns change over time).

**Figure 3:**
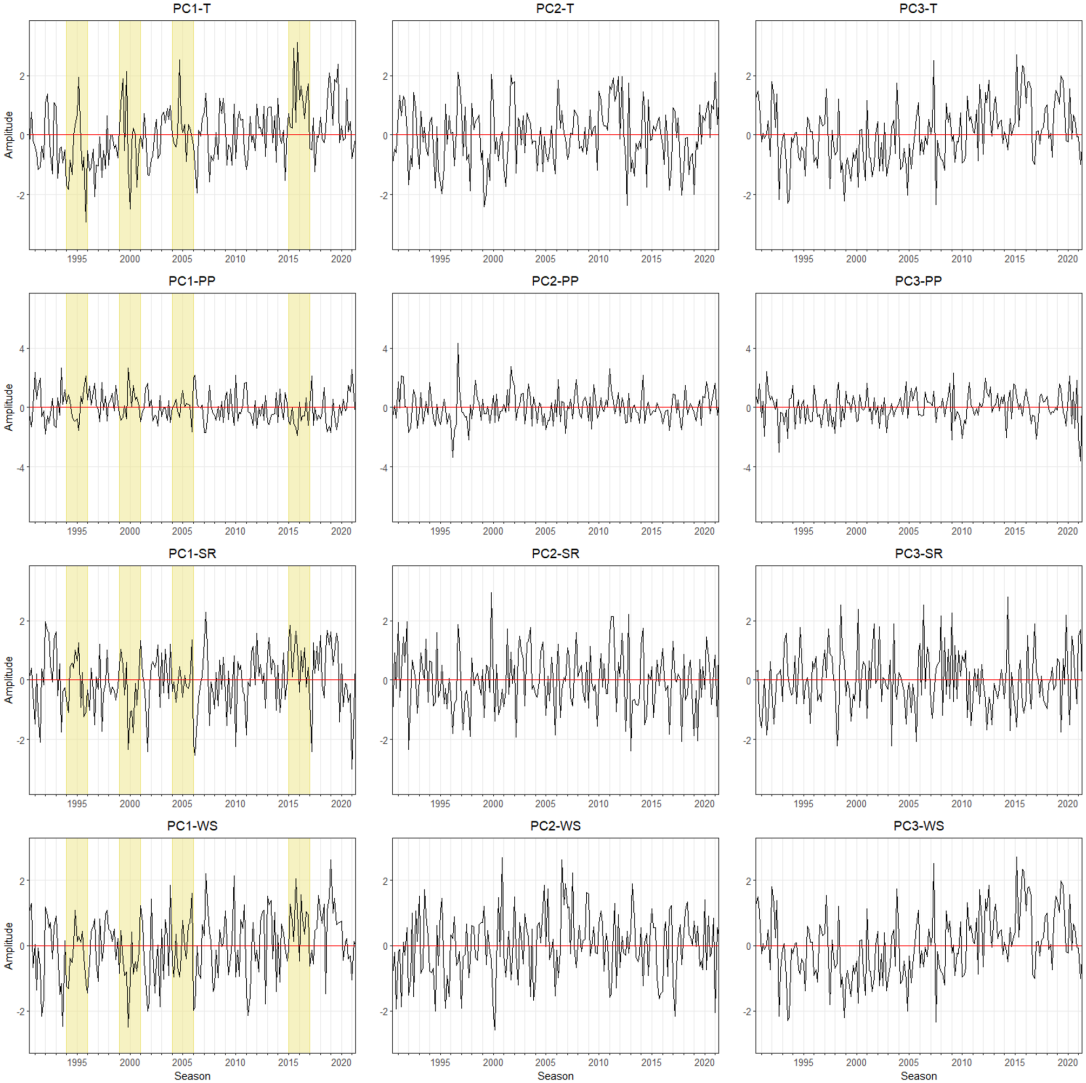
Monthly time series of growing season patterns (Principal components, PC) corresponding to EOF-1, EOF-2, and EOF-3 for the **Free State** province (see Fig. 2). Strong positive or negative anomalies of the dominant mode are highlighted (extreme events). The x-axis ticks indicate the beginning period of growing season. The abbreviations of agrometeorological data (T, PP, SR, WS) refer to Table 1.

The derived modes of agrometeorological variables in Free State can be seen in Fig. 2. The first EOF pattern of T (EOF-1) explains almost 80% of the variance. Positive loadings across Free State indicate a spatially uniform variation in T, in other words, there are significant no opposite (dipole) patterns. The corresponding temporal pattern (PC1-T) indicates that the leading spatial pattern captures years with extremely high temperatures (1994–1996, 1999–2001, 2004–2006, and 2015–2017) (Fig. 3). This indicates corresponding heat events were affecting the complete Free State province area. The same is true for the first EOF mode of PP, SR, and WS with rather low amplitudes of PC1-PP, PC1-SR, and PC1-WS during high-temperature events (Fig. 3). Contrastingly, the second mode (EOF-2, explaining about 8 to 16% of total variance), shows more pronounced spatial patterns which can be distinguished by having positive and negative loading across Free State. EOF-2 loadings of the T and SR anomalies are positive in the northeastern and rather negative in the southwestern regions. Despite the fluctuation of T, the corresponding time series pattern (PC2-T) does not show a significant extreme event. The second EOF mode (EOF2) of PP and WS indicate an opposite pattern where the positive loadings are occurring mostly in the southwestern region. The corresponding time series pattern (PC2-PP) shows a peak between 1996 and 1998, which relates to a time period with above average rainfall amounts. PC2-SR indicates a high amplitude between the season 1999 and 2001. In the same year, PC2-WS has an opposite pattern where it shows the lowest amplitude for the last 31 years. The third mode (EOF-3) of T is related to variations in the northern part and it explains 6.7% of the total variance. The third mode of PP and SR shows positive loadings in the southwestern area which represents 7.4% and 8% of the total variance, respectively. For WS, positive loadings of EOF-3 show a pronounced pattern with the highest values in the central part and it extends towards the northwestern region. The corresponding temporal pattern PC3-T, PC3-SR, and PC3-WS indicate a continuous abrupt jump pattern. PC3-PP shows the lowest amplitude for the last 31 years which is in the year 2020, meaning low precipitation was occurring during this year.

**Figure 4:**
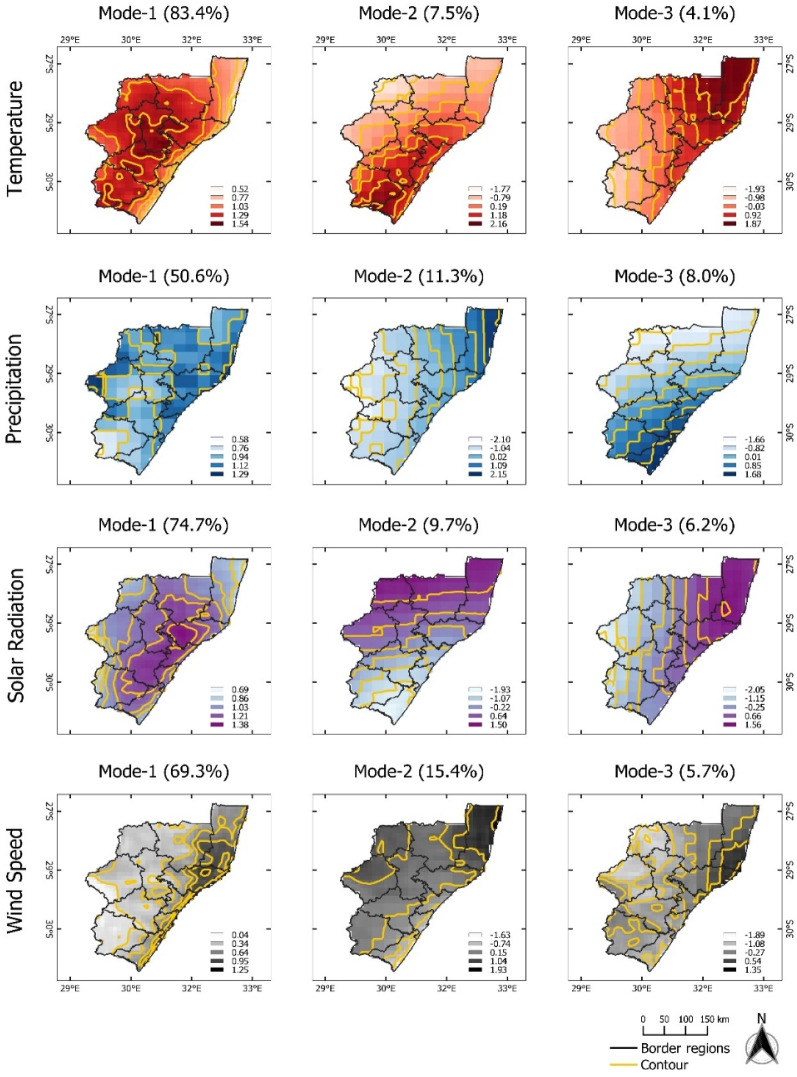
Map pattern of factor loadings for the first three modes (columns) derived from an EOF analysis for annual temperature, precipitation, solar radiation, and wind speed anomalies (rows) observed in the **KwaZulu-Natal** during the maize growing season of 1990/91–2020/21. Data for the maize growing season was used (cf. “Research data” section). Contour as for Fig. 2.

**Figure 5:**
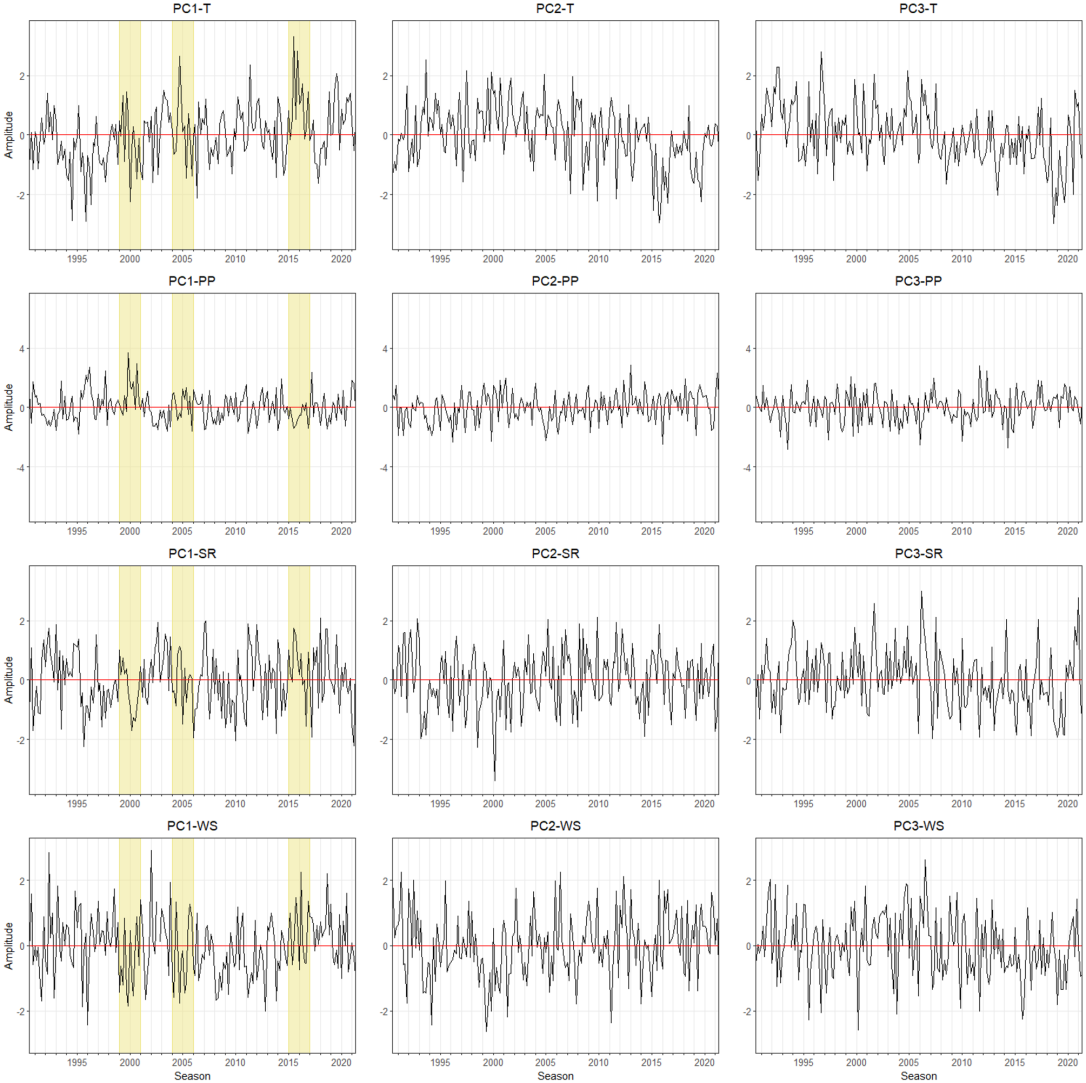
Monthly time series of growing season patterns in maize growing season (principal components, PC) corresponding to EOF-1, EOF-2, and EOF-3 for the **KwaZulu-Natal** province (see Fig. 4). Strong positive or negative anomalies of the dominant mode are highlighted (extreme events). The x-axis ticks indicate the beginning period of growing season. The abbreviations of agrometeorological data (T, PP, SR, WS) refer to Table 1.

For the KwaZulu-Natal province, the dominant mode (EOF-1) of T, PP, SR, and WS explains more than 50% of the total variance. There are no opposite positive and negative loading, indicating homogenous variation across the region (Fig. 4). The corresponding temporal pattern of T (PC1-T) shows high amplitudes of T in 2004–2006 and 2015–2017 (Fig. 5). On the other hand, PC2-T and PC3-T show low amplitudes between 2015 and 2020. Furthermore, PC1-PP illustrates high amplitudes between 1999 and 2001, revealing high PP occurred during the growing season. PC1-SR displays no significant abrupt change. PC1-WS demonstrates high amplitudes during 1990–1993 and 2001–2003, and low amplitude in 1995–1997. Dipole patterns can be observed for EOF-2, where EOF-2 of T captured the positive loading in the southwestern area. Positive loadings for PP, SR, and WS are mostly located in the northern and northeast coastal areas. Their corresponding temporal patterns PC2-PP and PC2-WS are formed in the same fluctuation range, except for PC2-PP, which shows a high jump between 2013 and 2014. For PC2-SR, the lowest amplitude can be identified in the season of 1999–2001. EOF-3 of T, PP, SR, and WS explain 4–8% of the total variance with positive values at the coastal areas gradually changing to negative values towards inland areas. The PC3-T temporal pattern reveals decreasing pattern T gradually from 2010 to 2020. PC3-SR and PC3-WS demonstrate similar high amplitudes patterns between 2006 and 2008. At the same time, low amplitude PC3-PP can be identified. PC3-PP shows high precipitation in subsequent season (2011–2013).

**Figure 6:**
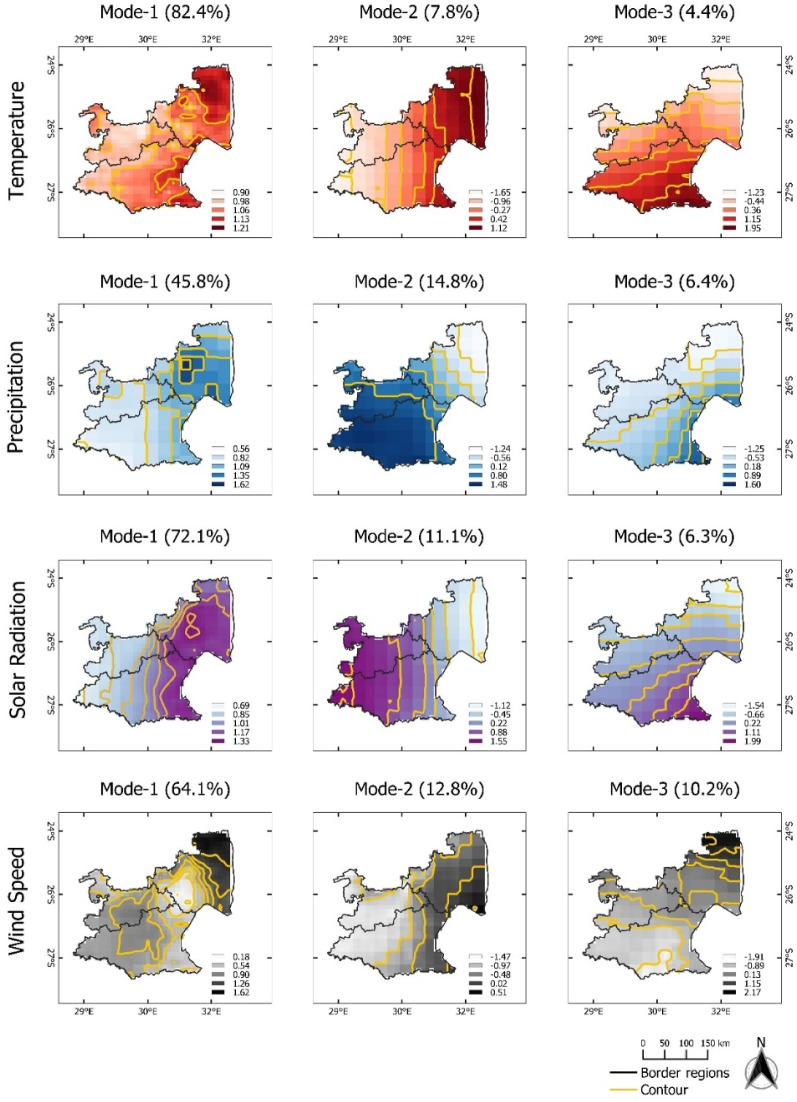
Map pattern of factor loadings for the first three modes (columns) derived from an EOF analysis for annual temperature, precipitation, solar radiation, and wind speed anomalies (rows) observed in the **Mpumalanga** province during the maize growing season of 1990/91–2020/21. Data for the maize growing season was used (cf. “Research data” section). Contour as for Fig. 2.

**Figure 7:**
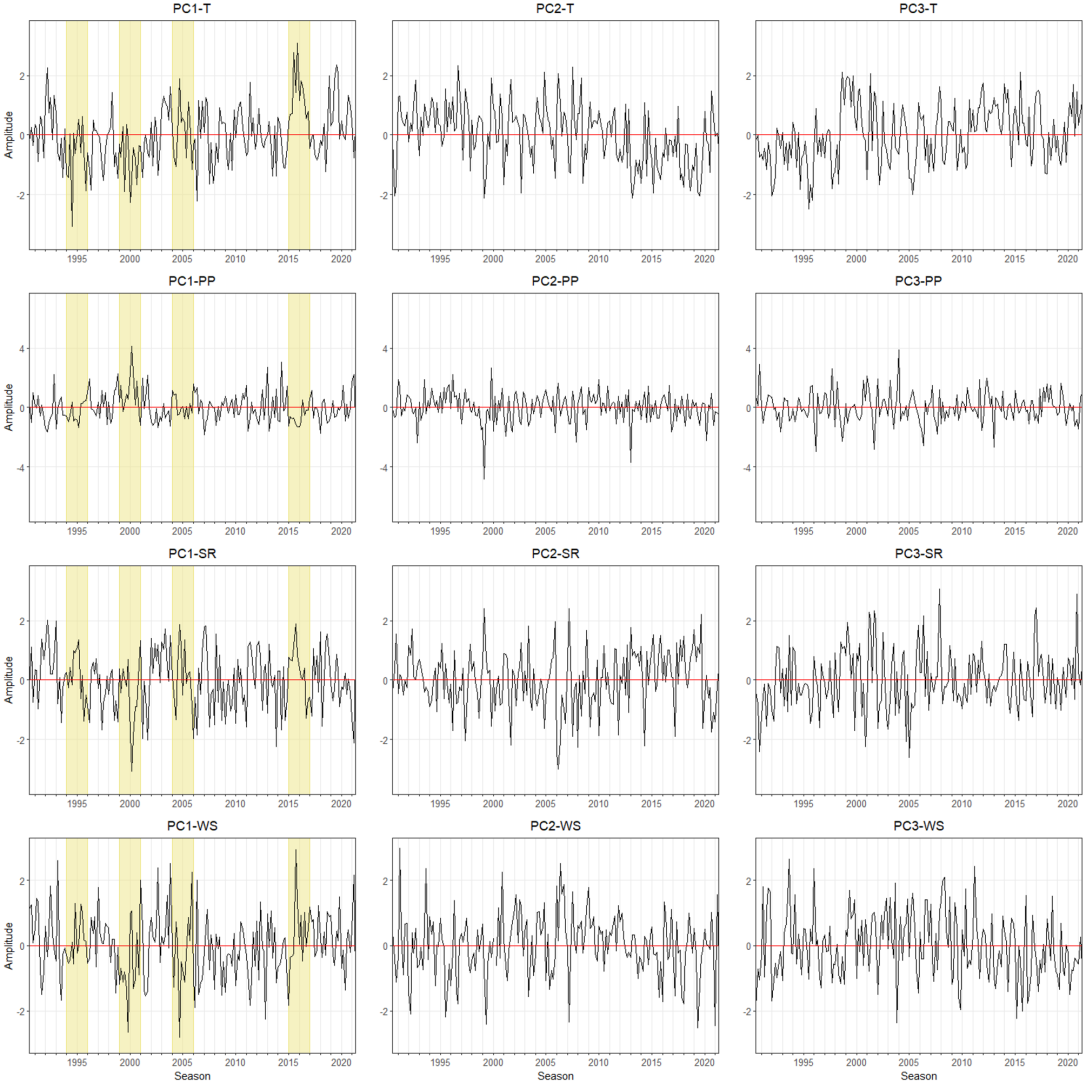
Monthly time series of growing season patterns (principal components, PC) corresponding to EOF-1, EOF-2, and EOF-3 for the **Mpumalanga** province (see Fig. 6). Strong positive or negative anomalies of the dominant mode are highlighted (extreme events). The x-axis ticks indicate the beginning period of growing season. The abbreviations of agrometeorological data (T, PP, SR, WS) refer to Table 1.

The leading mode (EOF-1) of T, PP, SR, and WS Mpumalanga explains 82%, 45%, 72%, and 64% of the total variance, respectively (Fig. 6). Given the positive loading across all regions, the variability of spatial patterns spread homogenously. The corresponding temporal patterns PC1-T and PC1-WS show a pronounced peak in 2015–2017 (Fig. 7). The highest amplitude of PC1-PP occurred between 1999 and 2001 which corresponds with decreased amplitudes for PC1-SR and PC1-WS. Similarly, PC1-WS demonstrate low amplitude in the season 2004 and 2006. EOF-2 indicates clear spatial patterns: positive loading of T and WS prevail in the northeastern, whereas positive loading of PP and SR are isolated in the southwestern areas. The corresponding temporal pattern in T (PC2-T) does not show significantly high amplitudes. For PC2-PP, a significant depth can be recognized between 1999 and 2013. An aburpt patterns between PC2-SR and PC2-WS can be identified between 2005 and 2007 where PC2-SR has the lowest value and PC2-WS demonstrate high amplitude. The highest WS (PC2-WS) occurred in the 1990 growing season. EOF-3 mode of T, PP, and SR have a similar spatial pattern, where negative loadings dominate in the northern regions, meanwhile for WS with negative loadings is influencing in the Southern area. The corresponding temporal patterns of PC3-T, PP, and SR are characterized by a pattern below average from 1990 to 1998. During the same time, PC3-WS demonstrates strong wind anomalies. In addition, PC3-PP illustrates multiple low precipitation events, with the lowest negative anomaly in the 1996 growing season.

**Figure 8:**
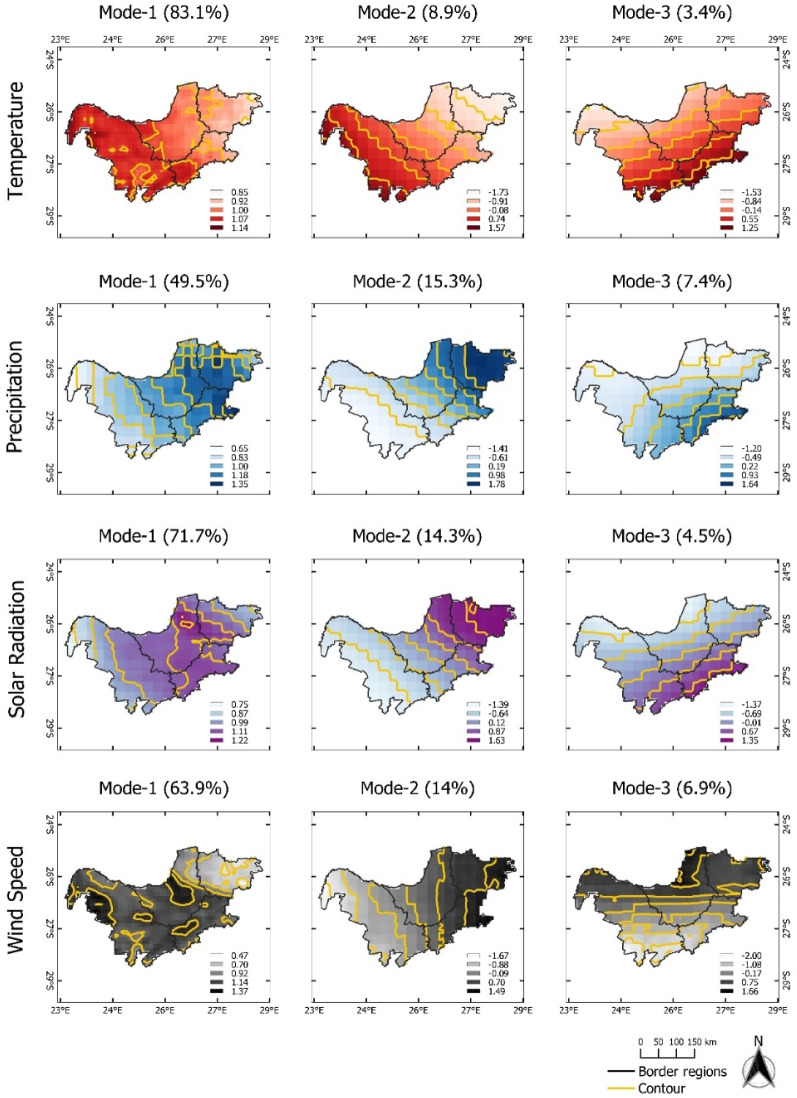
Map pattern of factor loadings for the first three modes (columns) derived from an EOF analysis for annual temperature, precipitation, solar radiation, and wind speed anomalies (rows) observed in the **North West** during the maize growing season of 1990/91–2020/21. Data for the maize growing season was used (cf. “Research data” section). Contour as for Fig. 2.

**Figure 9:**
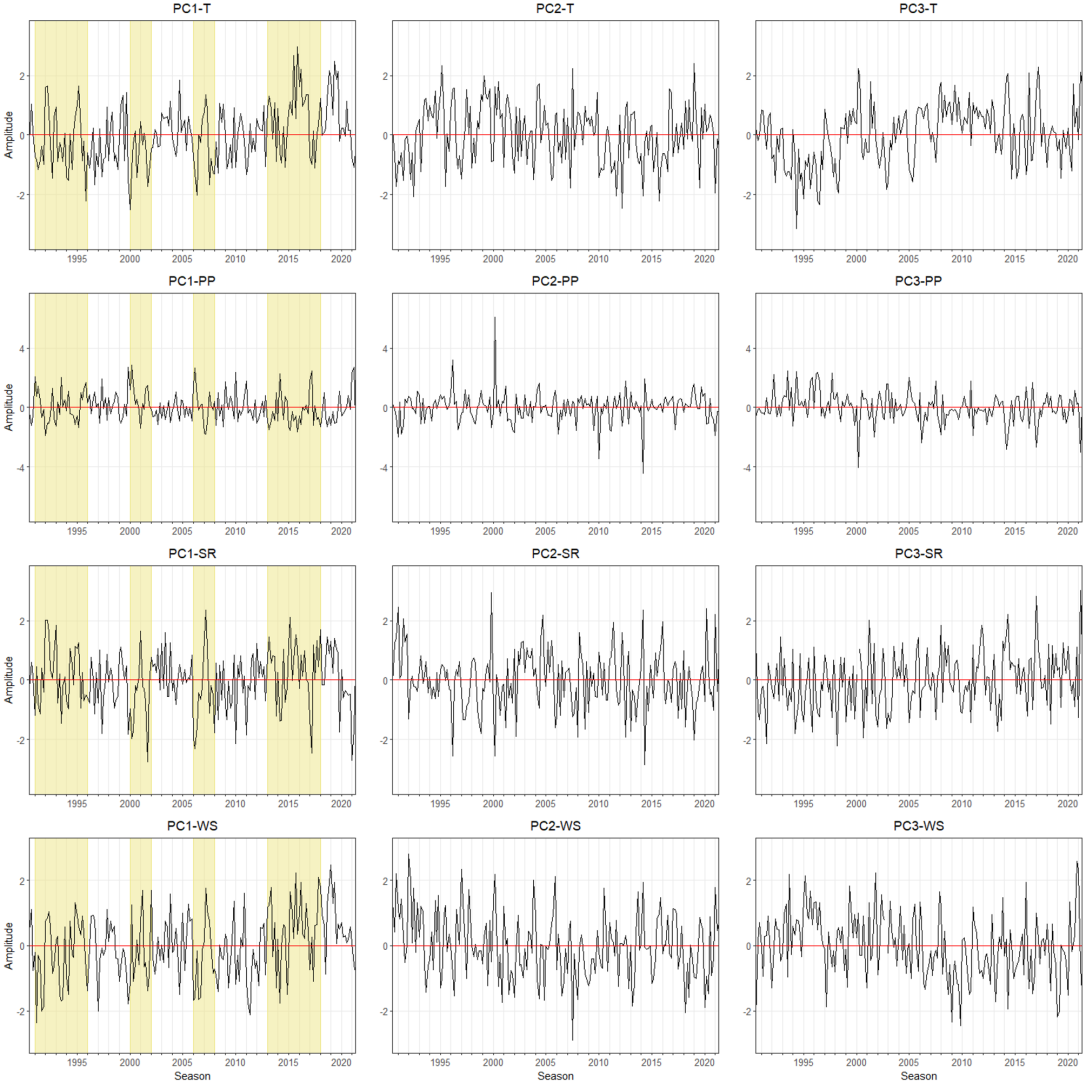
Monthly time series of growing season patterns (principal components, PC) corresponding to EOF-1, EOF-2, and EOF-3 for the **North West** province (see Fig. 8). Strong positive or negative anomalies of the dominant mode are highlighted (extreme events). The x-axis ticks indicate the beginning period of growing season. The abbreviations of agrometeorological data (T, PP, SR, WS) refer to Table 1.

For the North West province, the first spatial mode of each variable explains more than 50% of the total variance. As for the other provinces, there are no significant dipole patterns in EOF-1, indicating homogenous variation across the region (Fig. 8). Corresponding temporal patterns for temperatures (PC1-T) indicate a high temporal variability, with highest peaks between 2015 and 2017 (Fig. 9). The temporal pattern of the first mode for precipitation (PC1-PP) suggests positive precipitation anomalies for the periods 2000–2002, 2006–2008, and 2013–2018. At the same periods, PC1-SR has the lowest amplitude. PC1-WS does not show significant high and low spikes. EOF-2 of T shows a dipole pattern with positive loadings is concentrated in the southwestern regions whereas the second mode for PP, SR, and WS have positive and negative loadings in northeastern and southwestern regions, respectively. PC2-PP captures a single peak in 1995–1997 and 1998–2000, and a pronounced negative anomaly in 2013–2015. The corresponding temporal pattern of PC2-SR and PC2-WS show positive anomalies in 1999–2001 and 1991–1993, respectively. Furthermore, PC2-WS has the lowest amplitude between 2007 and 2008. EOF-3 demonstrates positive loadings for T, PP, and SR in the southeastern part, whereas for WS positive loadings of EOF-3 prevail in the northern regions. PC3-T is characterized by a pronounced negative anomaly in the season 1993–1995. The positive amplitude of PC3-SR and PC3-WS tend to increase gradually over the last 10 years. In contrast with PC3-PP, the negative amplitude is more pronounced from the season of 2000 until 2020.

## Discussion

Investigating trends, spatial and temporal patterns of agrometeorological variables and their relationship with crop yield is essential for understanding and monitoring the impact of climate change on agroecosystems and predicting future variability in crop production. To our best knowledge, the recently released reanalysis AgERA5 data set has not yet been assessed for studying the impact of climate change on historical maize production in the major maize-producing provinces of South Africa and for analyzing spatiotemporal patterns of agrometeorological during maize growing seasons.

Our findings show that there was an increase in temperatures (T) for both annual data and data limited to maize growing seasons. Analyses revealed significant positive trends in annual T at the rate of 0.03–0.04 °C per year. These trends are lower compared with those reported by Akanbi et al.^36^. They reported that the interannual temperature in the Vaal Catchment which is located along Mpumalanga, Gauteng, North West, Free State, and Northern Cape provinces increased by 0.47 °C between 1989 and 2018. If limiting data sets to the maize growing season, trends were lower compared with annual trends for KwaZulu-Natal and Mpumalanga (0.02 °C per growing season). Results align with van der Walt and Fitchett^37^. By utilizing climate data from weather stations across South Africa, they found an annual increase in maximum temperature of 0.02 °C per year between 1960 and 2016. Furthermore, we found no significant trends in precipitation (PP), except for annual PP in the Free State province. This agrees with a study by Kruger^38^ who examined data from 138 rainfall stations in South Africa over the period 1910–2004. The author reports that there was no significant trend in annual precipitation for most stations. Similarly, Murungweni et al.^39^ who studied data from 8 rainfall stations in the Limpopo province of South Africa between 1950 and 2016, report non-significant trends in annual and seasonal rainfall for station-specific available observation periods (except for one station). However, while the high interannual variability of annual precipitation data might mask trends, quantile regression analyses, indicate the presence of statistically significant up- and downwards trends.

Solar radiation data (SR) did not show significant trends. This could be related to the observation data used for the reanalysis model showing the accumulation of aerosols in the atmosphere which scatter direct solar radiation^40,41^. Furthermore, the fluctuation between clear and cloudy periods might offset or make it difficult to detect potential positive trends^42^. Our results are consistent with Stanhill and Moreshet^43^. Based on their observation data from Keetmanshoop (Namibian desert) from 1957 to 1981, SR did not show a significant linear or quadratic trend. In addition, it is to be expected that global horizontal solar irradiance remains rather unchanged until 2050^44^.

We detected a significant increase in annual WS for the Free State, KwaZulu-Natal, and North West provinces. For WS during the maize growing season (austral summer), only the data from the Free State province showed a significant upward trend. These trends might be due to the strong movement of south-eastern trade winds in summer over northeastern regions (Limpopo province) toward Free State^45^. Contrasting with our annual observations, based on observations from 19 weather stations for the period 1995–2014, Wright and Grab^46^ reported a decline in WS for the Cape province, thus, for the southernmost province of South Africa, especially for the coastal zones. Nchaba et al.^47^ also confirmed that there is a reduction of wind speed with an average of 0.7 and 1.3 ms^−1^ at 10 m and 850 hPa over the study period of 1980–2015, respectively. The discrepancy in absolute trends might be related to differences in the observation period: at the global scale, observations indicate that decreasing trends in wind speed have reversed since about 2010^48^.

We found significant negative correlations between temperature and maize yield in Free State and North West. Increasing temperatures can disturb silking date^49^, lower ovule fertilization^50^, as well as induce kernel abortion^51^. This would have a direct impact on kernel development and lead to lower yield. In contrast with precipitation, we found significant positive correlations between precipitation and maize yields except for KwaZulu-Natal. This is consistent with the results of Hadisu Bello et al.^52^ showing positive correlations between yield and precipitation for the Free State province. Because maize production in South Africa heavily relies on summer precipitation, less precipitation has a significant negative impact on maize yield^53^. Maize production in South Africa increased 11% during La Niña which is known as a wetter condition^54^. However, excessive precipitation events over longer time periods could also further negatively impact maize yields, especially in conjunction with poorly drained soils and erosion^55^. Poorly drained soil creates the condition that promotes waterlogging which could decrease photosynthesis capacity and inhibit plant growth^56,57^, Water erosion could cause soil and nutrient loss and thereby reduce maize growth and development^55,58^. According to Li et al.^59^, excessive rainfall can reduce maize yield up to 34%.

Our finding on the negative correlation between SR and yield contrasts with studies indicating positive relations^60^, highlighting that the interaction between multiple parameters (heat, precipitation events, wind speed, and solar radiation) has to be considered under field conditions. Besides droughts or extreme precipitation events, also increased wind speeds can lead to yield reductions. As might have been expected from our finding that wind speed shows a negative correlation with maize yield. Considering the increasing temperature trend with the strong wind, transpiration will be increased^61^. This could potentially lead to a high risk of drought stress. Another factor resulting in yield reduction by wind is stalk lodging. The strong force of the wind (abiotic factor) can snape the stalk, which causes the physical collapse of the canopy^62^. Flint-Garcia et al.^63^ mentioned that stalk lodging by biotic (pathogens and insects) and abiotic factor (wind) is accountable for 5–20% of maize yield losses worldwide. The authors acknowledge that the spatial distribution and total area of maize production, as well as management techniques and cultivar choices, changed throughout the observation period and might have affected the relation between yields and agrometeorological data.

The EOF analysis highlighted that the variation of agrometeorological data differs among provinces. The results from EOF analysis have to be interpreted carefully^64^. The variability (dipole pattern) of wind speed of the second and third modes was found over northwestern regions of Free State and in the eastern part of North West. This could be attributed to strong wind spiraling away from the high-pressure area over the South Indian Ocean, passing over Free State, and curving in the northern area of Free State. Additionally, this wind moves the warm air masses. It meets with cool dry air masses from the southwest which flow toward the inland and form thunderstorms (moisture boundary) along with Free State and North West. Hence, precipitation patterns in the second and third modes was dominant in the southwestern areas of Free State and the northerly areas up to the northern part of North West province. We also observed wind speeds pattern in the eastern region of KwaZulu-Natal and Mpumalanga. A possible explanation for this is the strong south-eastern trade wind that passes over KwaZulu-Natal and Mpumalanga towards Limpopo province^45^.

The predominant positive loading precipitation of Mode-1 in all regions indicate that the daily variability of precipitation was homogenous across the eastern part of South Africa with similar spatial patterns for extreme precipitation events^65^. Our findings on a dipole pattern in precipitation over eastern South Africa can be explained by the convergence of moist air originating from the Indian Ocean and tropical southern Africa^66–68^. A dipole pattern of temperature was detected in the eastern regions of South Africa (Free State, KwaZulu, and Mpumalanga). This can be explained by the warm Agulhas current on the east coast of South Africa during summer. The current release large amounts of heat into the atmosphere, thereby affecting eastern regional temperatures^69^.

Based on the leading time-series pattern of all regions, we observe extreme temperature events associated with low precipitation in 2015–2017. The combination of these events induced three consecutive dry years in South Africa^70^. During these years, precipitation in south western regions decreased by approximately 30% up to 50% below the long-term average rainfall^71^. This drought event was recorded in 2016 as the result of El Niño event^72^, which had a negative impact on the regional reservoir availability which decreased from 97% in 2014 to 38% in 2017^70^. We also identified multiple low amplitude of precipitation in 1992, 2007, and 2018 in the complete study area. The low precipitation in 2007 was the event that responsible for crop failures and led to food insecurity across the study regions and including the neighboring country Lesotho^73^. According to Masupha and Moeletsi^74^, in the near future (2020/21–2036/37) and far future (2055/56–2089/90), the reduction of soil moisture will be more pronounced which will have negative impact on rainfed maize performance in the Luvuvhu river catchment area, north-eastern part of South Africa.

Our finding on multiple peak signals of precipitation could be associated with La Niña event. The influence of La Niña on eastern South Africa regions is characterized by the above average precipitation^75^. Heavy precipitation events in 1999–2001 is also confirmed by Bellprat et al.^76^. This high precipitation event was the result of the tropical cyclones ‘Connie’ and ‘Eline’ in 2000 over Southern Africa. The strong wind in 1991–1993 and 2015–2017 was highly driven by thunderstorms and extratropical cyclones (the passage of cold fronts) across South Africa^45^.

Combining the observations on maize yields, their correlation with agrometeorological data, and the dominant spatiotemporal patterns for each province allows for identifying the main factors influencing maize yield fluctuations between 1990/91 and 2020/21. Based on the temperature (T) and wind speed (WS) events captured by the PC that corresponds with the leading mode for the Free State province, maize yield fluctuations in this area were likely driven by extreme temperature events and strong southeastern trade wind. As reported by Mbiriri et al.^77^, the impact of El Niño in Free State is more intense at the low elevation level rather than at the high elevation. This suggest that drought event (El Niño) will have a devastating impact on maize production especially most of maize field (crop land) are located at low land of Free State. For the North West province, the results imply that the frequency of extreme events of T, PP, SR, and WS were the main factors for the temporal variability of maize yield. This indicates that the maize production in corresponding regions might be particularly affected by future climate change.

For the KwaZulu-Natal, the leading mode is also characterized by positive trends in T and WS and by extreme events (strong positive anomalies) for T, PP, and WS. However, maize yield was not influenced by agrometeorological growing season data. Findings, therefore, suggest that yields were rather driven by extreme events that are not captured by the growing season means or by non-climatic factors, such as technological progress or changes in agronomic management. Extreme positive T, PP, SR, and WS anomalies were also found for the leading mode of time series data for the Mpumalanga province. However, only growing season PP and SR showed significant positive and negative correlations with maize yields, respectively, thereby suggesting that changes in thunderstorms and tropical cyclones might be the primary factor for yield reductions. This implies that the subtropical southern Indian Ocean creating high rainfall associated with a solar radiation change in northeastern regions might have been the primary factor affecting the interannual variability and trends in maize yields. We suggest that further studies might be required to evaluate the interaction between different agrometeorological variables and their joint effect on crop yields. Our findings provide strong evidence for the negative impact of climate change on maize production in central maize production provinces of South Africa during the last decades. Therefore, maize production in South Africa will likely suffer greatly from climate change in the future. This trend will intensify unless agricultural management is adapted and measures are taken to slow down climate change.

## Conclusions

The main conclusions that emerge from this study are:

- Reductions in maize production in South Africa can be related to a consistent upward trend in temperatures at both annual (0.03–0.04 °C) and growing season scales (0.02–0.04 °C). Climate change likely induced a statistically significant reduction in precipitation in the Free State region at a level of 0.01 mm per year. The trend of solar radiation does not change for the last 31 years. Increasing wind speed annually was found in Free State, Kwazulu-Natal, and North West. The increasing wind speed growing season was observed in Free State at level 0.01 ms^−1^ per growing season.
- The main drivers for the variability of yields in Free State and North West were temperature, precipitation, solar radiation, and wind speed while agrometeorological variables cannot explain changes in maize yields for KwaZulu-Natal. Interannual variabilities of maize yield in Mpumalanga were driven by changes in precipitation and solar radiation.
- The leading modes of the variability of agrometeorological data further imply that most of the climate variabilities and extreme events uniformly affected the regions throughout the area. The variabilities of agrometeorological variables in major maize production provinces (eastern South Africa) were influenced by south Indian high pressure and the warm Agulhas Current. Temporal patterns resulting from EOF analyses were suitable for identifying years of extreme events (high and low amplitude) that correspond to different spatial patterns.

Overall, our ability to predict future crop production relies on having a deep understanding of agrometeorological trends and patterns. As many scientists have recognized the impact of climate change on crop production worldwide, more profound actions towards sustainable agriculture practices are urgently needed. Agriculture production needs to cope and adapt to changing climate conditions worldwide, so that we can maintain, if not increase, our crop production, particularly in regions that heavily depend on regional food production. Results from this study and similar research studies could be used to assist South Africa’s government in favor of policy development to prevent famine.

## Supplementary Information

Supplementary Information.

## Data Availability

The datasets used and/or analyzed during the current study are available from the corresponding author on reasonable request.
