# Supplementary material for: Spatial and temporal patterns of agrometeorological indicators in maize producing provinces of South Africa
Source: Sci Rep. 2022 Jul 15;12:12072. doi: 10.1038/s41598-022-15847-7 (PMC9287399; doi:10.1038/s41598-022-15847-7)
Supplement: Supplementary file 1 — Supplementary Information. [file 41598_2022_15847_MOESM1_ESM.docx]

**Spatial and temporal patterns of agrometeorological indicators in maize producing provinces of South Africa**

**Christian Simanjuntak^1,^*, Thomas Gaiser^1^, Hella Ellen Ahrends^2^, and Amit Kumar Srivastava^1^**

^1^Institute of Crop Science and Resource Conservation, University of Bonn, Katzenburgweg 5, 53115, Bonn, Germany.

^2^Department of Agricultural Sciences, University of Helsinki, Koetilantie 5, 00014, Helsinki, Finland.

^*^Email: simanjuntak_christ@yahoo.co.id

**Supplementary material**

Annual yield and total maize production area in South Africa from 1990/91─2020/21


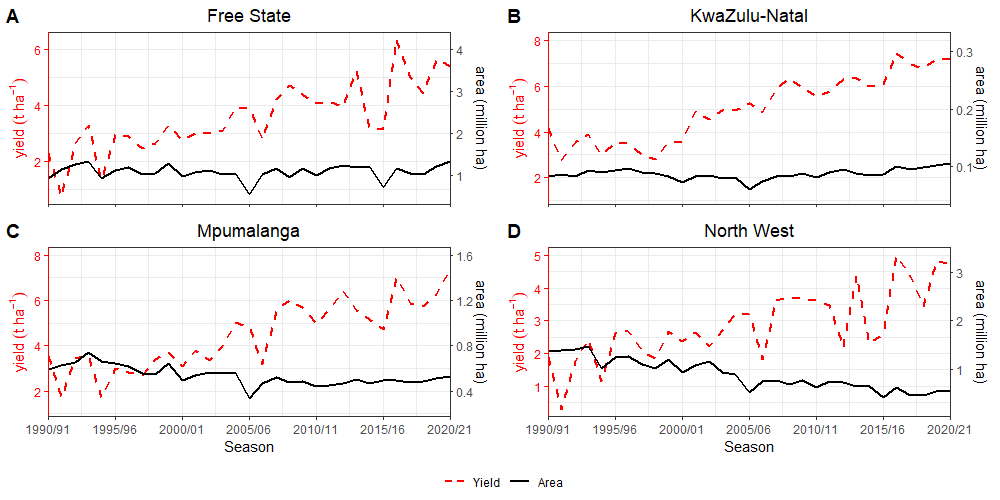


**Figure S1.** Annual maize yield (t ha^-1^) and total maize production area (million ha) for the provinces Free State, KwaZulu-Natal, Mpumalanga, and North West for growing season 1990/91 until 2020/21.

Total maize production in South Africa


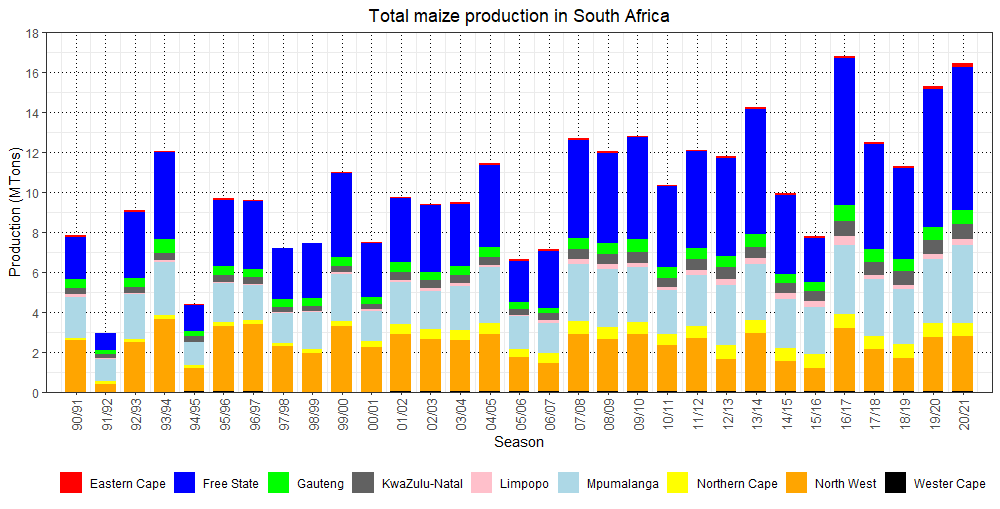


**Figure S2.** Total maize production in 9 provinces of South Africa.

Annual temperature from 1990─2020

**
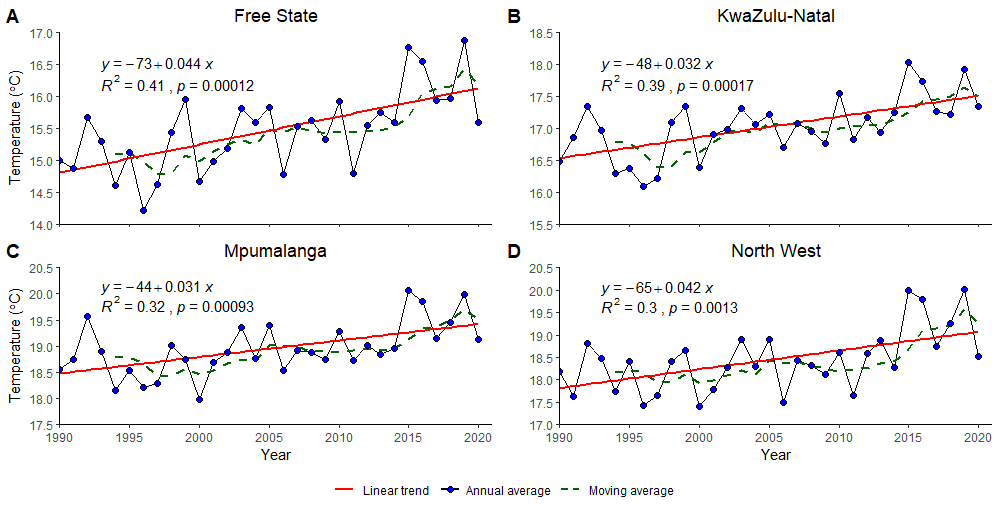
**

**Figure S3.** Annual temperatures between 1990 and 2020 and corresponding trends for the (a) Free State, (b) KwaZulu-Natal, (c) Mpumalanga, and (d) North West provinces. Linear regression lines for significantly increasing T values (cf. Table 2) are shown (solid red lines) and the 5 years moving average is indicated (dashed green line).

Growing season temperature from 1990/91─2020/21


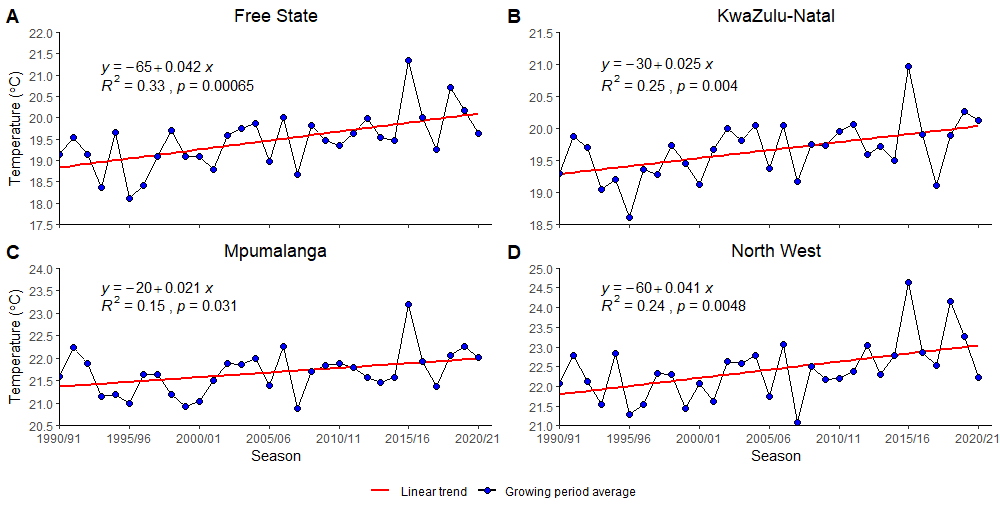


**Figure S4.** Maize growing season temperatures between 1990/91 and 2020/21 and corresponding trends for the (a) Free State, (b) KwaZulu-Natal, (c) Mpumalanga, and (d) North West provinces. Symbols as in Figure S3.


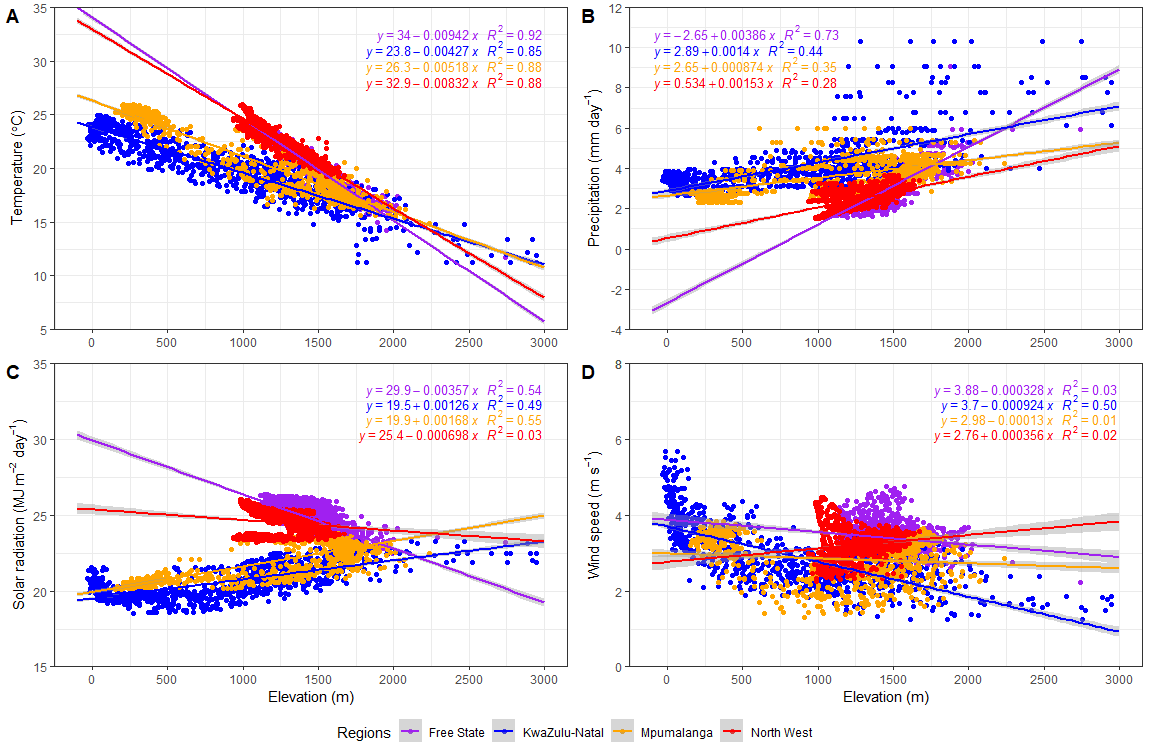
The relationship between agrometeorological and elevation

**Figure S5**. The regression analysis between (a) temperature, (b) precipitation, (c) solar radiation, (d) wind speed and elevation in Free State, KwaZulu-Natal, Mpumalanga, and North West during maize growing season 1990/91 to 2020/21. The straight line represents the linear fit and the grey band indicates the 95% prediction interval. Seasonal means of agrometeorological data are shown as dotted lines.

Land cover change in between 2015 and 2019

**
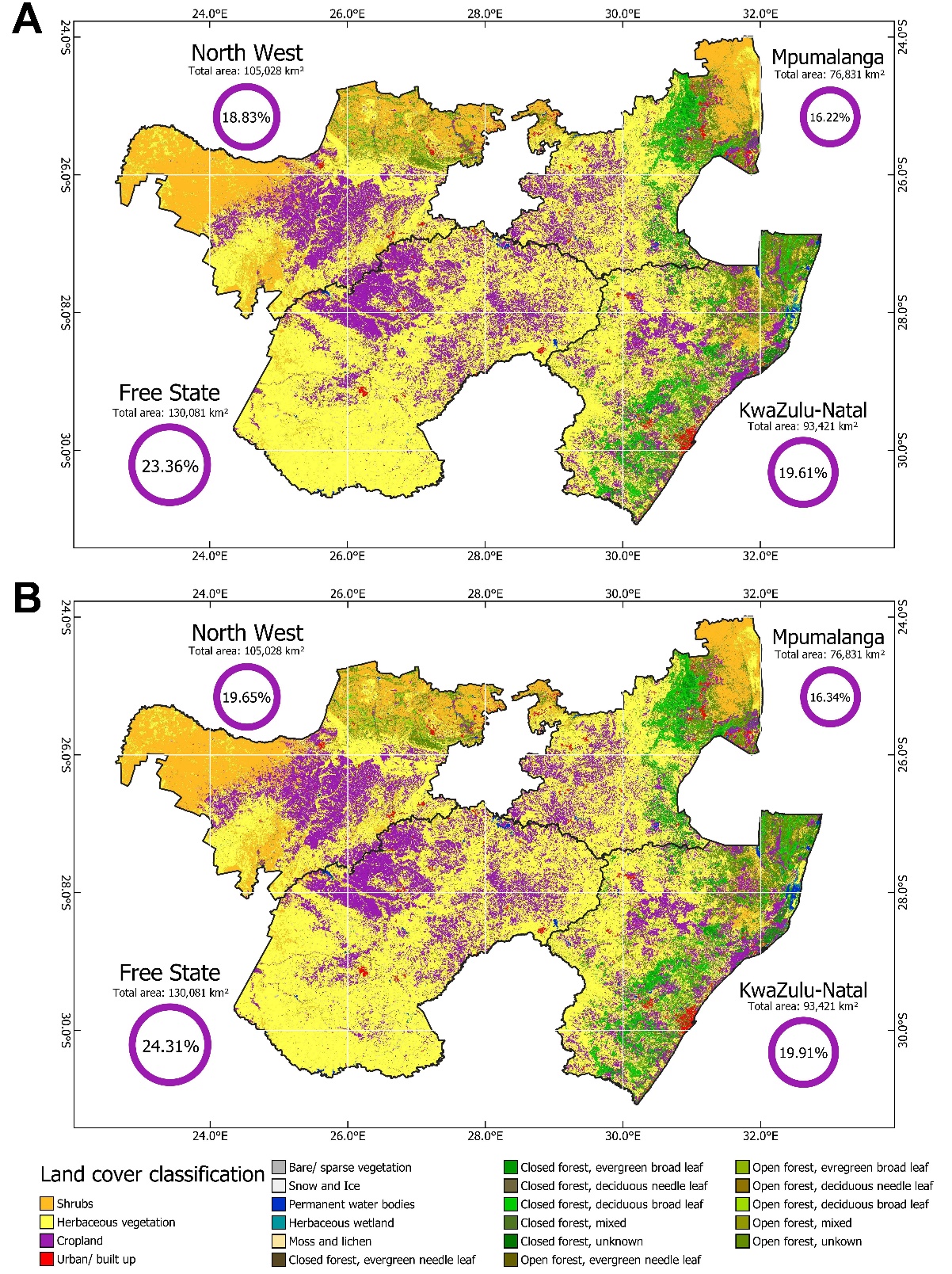
**

**Figure S6**. The land cover change of the major maize production provinces in South Africa between (a) 2015 and (b) 2019. The percentage represents the statistical ratio between cropland (purple color) and total land area in each province.

Land cover classification in 2019

**
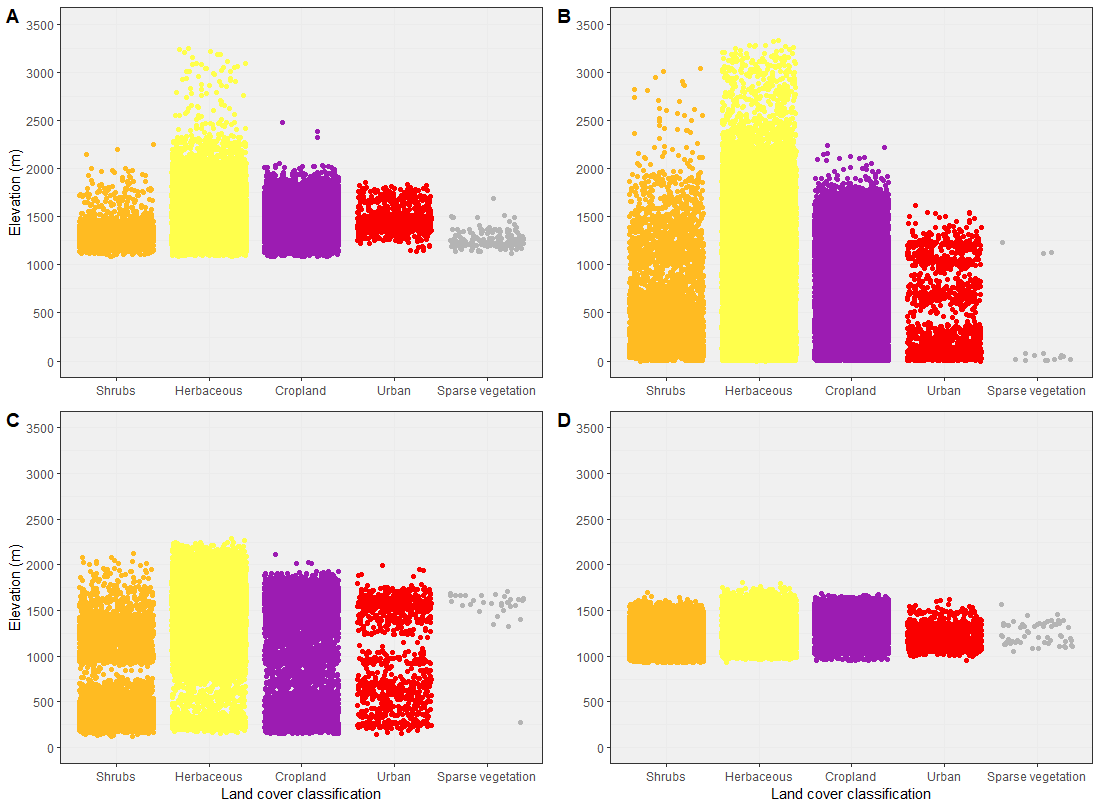
**

**Figure S7**. The distribution of land cover classification, namely shrubs, herbaceous, cropland, urban, and sparse vegetation in (a) Free State, (b) KwaZulu-Natal, (c) Mpumalanga, and (d) North west with respect to elevation in 2019.
